# ANXA2P2: A Potential Immunological and Prognostic Signature in Ovarian Serous Cystadenocarcinoma *via* Pan-Carcinoma Synthesis

**DOI:** 10.3389/fonc.2022.818977

**Published:** 2022-02-08

**Authors:** Yanna Zhang, Ting Du, Xiancheng Chen

**Affiliations:** ^1^ State Key Laboratory of Biotherapy/Collaborative Innovation Center for Biotherapy, West China Hospital, Sichuan University, Chengdu, China; ^2^ Noncoding RNA and Drug Discovery Key Laboratory of Sichuan Province, Chengdu Medical College, Chengdu, China

**Keywords:** ANXA2P2, pseudogene, immune characteristics, prognostic signature, ovarian serous cystadenocarcinoma

## Abstract

**Background:**

Although the effect of pseudogene ANXA2P2 on some tumors has been reported in a few literatures, the therapeutic potential and prognostic value of ANXA2P2 in ovarian serous cystadenocarcinoma (OV) have not been elucidated.

**Methods:**

The correlation for ANXA2P2 expression patterns to prognostic characteristics, tumor immune microenvironment, immune cell infiltration level, tumor mutation burden (TMB), tumor microsatellite instability (MSI), drug sensitivity, and pathway function enrichment were investigated in pan-carcinoma *via* TCGA and GTEx databases. Subsequently, the role of ANXA2P2 expression levels in the pathway enrichments and prognosis prediction in OV were further explored using weighted correlation network analysis (WGCNA) analysis, gene mutation analysis, and risk-independent prognostic analysis.

**Results:**

ANXA2P2 was frequently overexpressed in a variety of tumors compared with normal tissues. The correlation analysis for prognostic characteristics, tumor immune microenvironment, immune cell infiltration level, TMB, MSI, drug sensitivity, and pathway function enrichment revealed that ANXA2P2 expression patterns might deal a significant impact on the pathogenesis, development, and prognosis of various tumors. Then, GSVA, GSEA, WGCNA, gene mutation, and independent prognostic analysis for OV have indicated that high expression in ANXA2P2 could be mostly enriched in TNF-α signaling-*via*-NF-κB, epithelial-mesenchymal transition, apical junction, IL-6-JAK STAT3 signaling, etc., which were also proved to act as crucial factors on tumorigenesis, development, invasion, and metastasis. The mutation of TP53 (94%), TTN (24%), and CSMD3 (9%) in the biological process of tumor had been confirmed by relevant studies. Finally, the independent prognostic analysis demonstrated that ANXA2P2 expression in OV contributes greatly to the dependability of 3- and 5-year survival prediction.

**Conclusion:**

In summary, our findings might provide a helpful foundation for prospective explorative researches, afford new strategies for the clinical treatment, deal prognosis prediction, and give new hope for OV patients.

## Introduction

Ovarian serous cystadenocarcinoma (OV) is one of the most common epithelial malignant tumors of the female reproductive system, accounting for about 30% of all ovarian carcinomas (1, 2). Due to strong potential for invasion and metastasis, OV usually spreads beyond the pelvis when diagnosed (3, 4), thus, the prognosis of patients with advanced OV is very poor finally (5). Therefore, it is urgently needed to reveal the relevant pathogenesis and find novel potential targets with great clinical significance for treatment and prognosis evolution of OV.

ANXA2P2 (annexin A2 pseudogene 2, also known as ANX2L2, ANX2P2, or LPC2B), is one of three pseudogenes of annexin A2 (ANXA2) that has recently been shown to be aberrantly transcribed in various tumors. It has been found that ANXA2P2 pseudogene maps to chromosome 9p13 (6). Based on previous experience and literature reports indicating that the changes of ANXA2P2 were consistent with those of ANXA2 in multiple pathophysiological processes, the function of pseudogene would better be analyzed together with its parental gene. Simultaneously, high expression of ANXA2 had been confirmed to play a pivotal role in tumor cell adhesion, proliferation, apoptosis, invasion, and metastasis (7–9). It has been found that ANXA2 could overexpress in multitumors, including ovarian cancer, breast cancer, and glioma and enhance the expression of plasminase receptor on the surface of tumor cells (10–13). ANXA2 was also involved in DNA synthesis and cell proliferation by regulating the c-myc function (14). One of the main functions of the protein encoded by the c-myc gene is to promote cell proliferation by activating relevant transcription factors (15–17). Some other studies had also suggested that ANXA2 could increase the activity of DNA polymerase and thus promote the invasive growth and metastasis of tumor cells to surrounding tissues (18–20). However, it is unclear whether its pseudogene ANXA2P2 also has the same predictive value, and its clinical significance and biological function in OV were unclear as well.

With the development of biological science and technology, the understanding of pseudogenes to public has reached a new level from “gene fossil junk genes” (21, 22). Pseudogenes were considered nonfunctional genes because of loss of protein coding ability or without expression in cells (23). However, recent studies have found that some of them might deal an important role in regulating parental genes, and even some pseudogenes could be transcribed into RNA (24, 25). Some pseudogenes are now considered a potential tumor suppressor gene or oncogene and played a crucial role in the occurrence and development of many pathophysiological processes (22, 23). Thus, in our study, we would comprehensively analyze the relevance between ANXA2P2 expression patterns and prognostic characteristics in pan-carcinoma firstly. Additionally, the association with tumor immune microenvironment, immune cell infiltration level, tumor mutation burden, or tumor microsatellite instability to ANXA2P2 would be investigated so as to preferably understand whether ANXA2P2 expression levels and patterns were relevant to immunological signature and the prognosis in various cancers secondly. Next, combined with drug sensitivity analysis, pathway function enrichment would be also performed to validate the critical role of ANXA2P2 in multitumors. Since ANXA2P2 had not been reported in OV, weighted correlation network analysis (WGCNA) analysis, gene mutation analysis, and risk independent prognostic analysis would be further performed based on ANXA2P2 expression patterns to further substantiate its effect on the immune microenvironment and prognosis assessment of OV, so as to be able to provide a novel perspective for revealing the pathogenesis and looking for novel potential targets on treatment and prognosis judgement of OV.

## Materials and Methods

### Data Acquisition and Related Difference Collection

The Cancer Genome Atlas Program (TCGA) database (https://portal.gdc.cancer.gov/) is currently the largest cancer gene information database, which consisted of gene transcriptome profiles, copy number variation, single nucleotide polymorphisms (SNP), and relevant clinical information. A total of 33 tumor-related data were downloaded from TCGA for subsequent analysis, including adrenocortical carcinoma (ACC), bladder urothelial carcinoma (BLCA), breast invasive carcinoma (BRCA), cervical squamous cell carcinoma and endocervical adenocarcinoma (CESC), cholangiocarcinoma (CHOL), colon adenocarcinoma (COAD), lymphoid neoplasm diffuse large B-cell lymphoma (DLBC), esophageal carcinoma (ESCA), glioblastoma multiforme (GBM), head and neck squamous cell carcinoma (HNSC), kidney chromophobe (KICH), kidney renal papillary cell carcinoma (KIRP), acute myeloid leukemia (LAML), brain lower-grade glioma (LGG), liver hepatocellular carcinoma (LIHC), lung adenocarcinoma (LUAD), lung squamous cell carcinoma (LUSC), mesothelioma (MESO), ovarian serous cystadenocarcinoma (OV), pancreatic adenocarcinoma (PAAD), pheochromocytoma and paraganglioma (PCPG), prostate adenocarcinoma (PRAD), rectum adenocarcinoma (READ), sarcoma (SARC), skin cutaneous melanoma (SKCM), stomach adenocarcinoma (STAD), testicular germ cell tumors (TGCT), thyroid carcinoma (THCA), thymoma (THYM), uterine corpus endometrial carcinoma (UCEC), uterine carcinosarcoma (USC), and uveal melanoma (UVM). Meanwhile, gene expression data of different tissues were obtained from the Genotype-Tissue Expression (GTEx) database (https://commonfund.nih.gov/GTEx). Combined with corrected TCGA and GTEx data, the expression differences of genes were then calculated in different cancers. Next, the Pearson correlation analyses were performed for pseudogene ANXA2P2 and its parental gene ANXA2 expression in pan-carcinoma. In addition, the correlation between expression and tumor stage was also investigated in various cancers.

### Associated Prognostic Analysis

The overall survival (OS) and progression-free interval (PFI) data of TCGA patients were downloaded from the UCSC Xena (https://xena.ucsc.edu/) database to further explore the relationship between ANXA2P2 expression and patient prognosis. The connection between the ANXA2P2 expression and the prognosis of patients, including OS and PFI in 33 types of cancer were examined using forest plots and Kaplan-Meier curves, which were evaluated with “Survival”, “forestplot”, and “SurvMiner” package. Univariate and multivariate Cox regression analyses were performed to evaluate the prognostic value for the age, grade, and ANXA2P2 in OV.

### Immune Cell Infiltration Analysis

RNA-seq data from 33 cancer patients were analyzed using the Cell-type Identification by Estimating Relative Subsets of RNA Transcripts (CIBERSORT) algorithm (26) to investigate the relative proportion of various immunocyte types and to inquire the relevance between ANXA2P2 expression and various immunocyte contents. Simultaneously, potential relationships between ANXA2P2 expression and immunomodulators (immunostimulators, immunoinhibitors, immune checkpoint, chemokines, and MHC molecules) were explored through the Tumor-Immune System Interactions Database (TISIDB) website (27). In addition, the relevance was also explored in regulators of usual tumor, such as TNF-α signaling *via* NF-κB, TGF-β signaling, hypoxia, pyroptosis, DNA repair, autophagy, and ferroptosis-related regulators.

### Drug Sensitivity Analysis

The CellMiner (https://discover.nci.nih.gov/cellminer/) database is based on a list of 60 cancer cells listed by the National Cancer Institute (NCI) (28, 29). These cell lines are currently the most widely used sample library of cancer cells for testing anticancer drugs. In this study, drug sensitivity data and ANXA2P2-related expression data were downloaded to explore the relationship between ANXA2P2 and sensitivity to common antitumor drugs through correlation analysis. *p* < 0.05 is considered statistically significant.

### Gene Set Variation Analysis

Gene set variation analysis (GSVA) is a nonparametric and unsupervised method for assessing the enrichment of transcriptome gene sets (30). Through the comprehensive scoring the concerned gene sets, changes in gene transcription were transformed into the pathway level changes to predict and judge the biological function of the samples. In our study, gene sets were obtained from The Molecular Signatures Database (Version 7.0), and the potential biological function changes of different samples were evaluated *via* comprehensively scored gene set using the GSVA algorithm.

### Gene Set Enrichment Analysis

The Gene Set Enrichment Analysis (GSEA) uses a predefined gene set to rank genes according to the degree of differential expression in the two types of samples, and then test whether the preset gene set is enriched at the top or bottom of the ranking table (31, 32). We compare the differences in signaling pathways between high and low ANXA2P2 expression groups and explore the potential molecular mechanisms of prognosis differences in various tumor using “cluster profiler” and “enrich plot” packages.

### Association of ANXA2P2 Expression With TMB and MSI

TMB is defined as the total number of somatic gene coding errors, base substitutions, insertions, or deletions detected per million bases (33). In our study, TMB was determined by calculating the variation frequency and number of variants/exon length of each tumor sample and dividing the nonsynonymous mutation sites by the total length of protein coding region. The MSI values of each TCGA patient were derived from previously published studies (34).

### Epigenetic Mutation Analysis in OV

The corresponding somatic alteration information of the OV were obtained from the TCGA dataset. The somatic alteration mainly contained Nonsense Mutation, Missense Mutation, Frame Shift Del, Frame Shift Ins, Splice Site, In Frame Del, In Frame Ins, and Multi Hit. The “maftools” and “ComplexHeatmap” R packages were employed to calculate and visualize the number of somatic mutations within every patient.

### Establishment the Nomogram Prediction Model for OV

A prognostic nomogram was constructed by using the “rms” R package (https://cran.r-project.org/web/packages/rms/) to evaluate the 3- and 5-year survival probability of OV patients, where age, grade, and ANXA2P2 were included as independent parameters. Next, the calibration curves were established to evaluate discrimination and calibration between the nomogram-predicted feasibility and observed survival probability.

### Statistical Analysis

All statistical analyses were performed using R software (version 4.0.2). Hazard ratios (HRs) and 95% confidence intervals were calculated using univariate survival analysis. Kaplan-Meier analysis was used to explore patient survival based on high or low levels of ANXA2P2 expression. All statistical tests were two sided, and *p* < 0.05 was considered statistically significant.

## Results

### Transcription Expression Level of ANXA2P2 in Pan-Carcinoma

**Figure 1:**
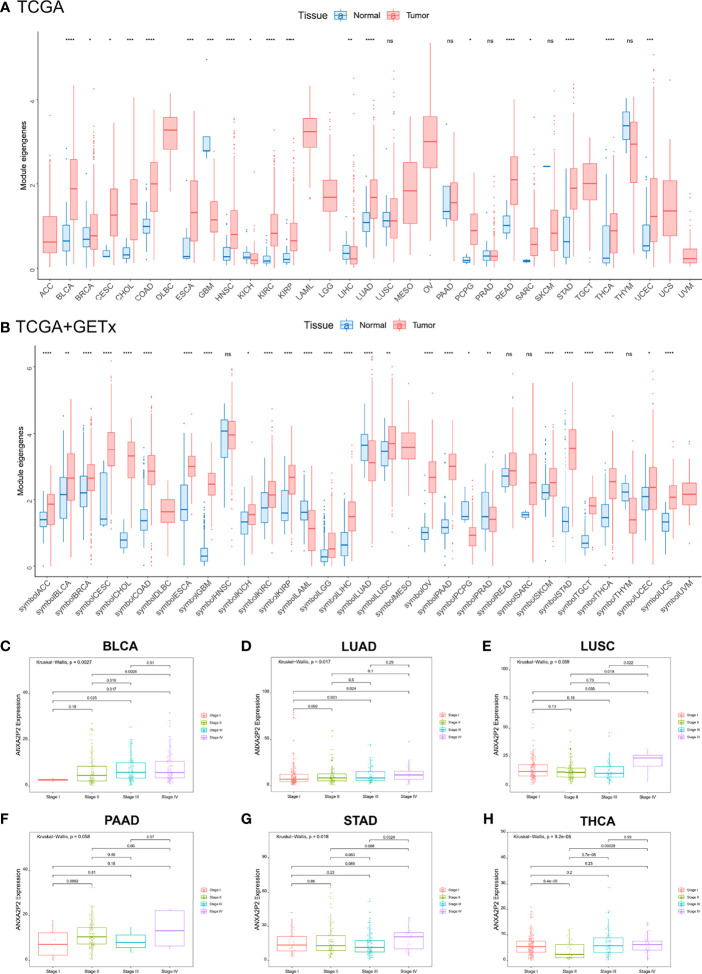
Differential transcriptional expression of ANXA2P2 and its correlation to tumor stages in pan-carcinoma. **(A)** The landscape of ANXA2P2 expression levels based on TCGA dataset. *P < 0.05, **P < 0,01, ***P < 0.001, ****P < 0.0001 and ns indicated no significant. **(B)** The landscape of ANXA2P2 expression levels based on TCGA and GTEx datasets; red represents tumor tissue, and blue represents normal tissue. Correlations between ANXA2P2 expression and tumor stages in **(C)** BLCA, **(D)** LUAD, **(E)** LUSC, **(F)** PAAD, **(G)** STAD, and **(H)** THCA.

Transcriptional expression landscapes of ANXA2P2 in 33 human cancers compared with normal tissues were obtained from TCGA or GTEx datasets. According to the TCGA transcriptome data, ANXA2P2 was significantly overexpressed in a variety of tumors compared with normal tissues, including BRCA, CESC, CHOL, COAD, ESCA, GBM, KICH, KIRC, KIRP, LIHC, LUAD, LUSC, STAD, THCA, and UCEC (**Figure 1A**). Simultaneously, the expression level of ANXA2P2 was determined by combining TCGA and GETx transcription data, and the upregulated ANXA2P2 expression was observed consistently in tumor tissues versus normal tissues in ACC, BLCA, BRCA, CESC, CHOL, COAD, ESCA, GBM, KICH, KIRC, KIRP, LGG, LIHC, LUSC, OV, PAAD, SKCM, STAD, TGCT, THCA, UCEC, and UCS (**Figure 1B**). These results indicated that ANXA2P2 expression levels were higher in most human tumors than in normal tissues. In addition, the correlations between ANXA2P2 and tumor stages were analyzed based on TGCA (**Figures 1C**–**H** and **S1**). It revealed that ANXA2P2 was related to the stages of a variety of tumors, involving BLCA (**Figure 1C**), LUAD (**Figure 1D**), LUSC (**Figure 1E**), PAAD (**Figure 1F**), STAD (**Figure 1G**), and THCA (**Figure 1H**).

### Associated Prognostic Analysis of ANXA2P2

**Figure 2:**
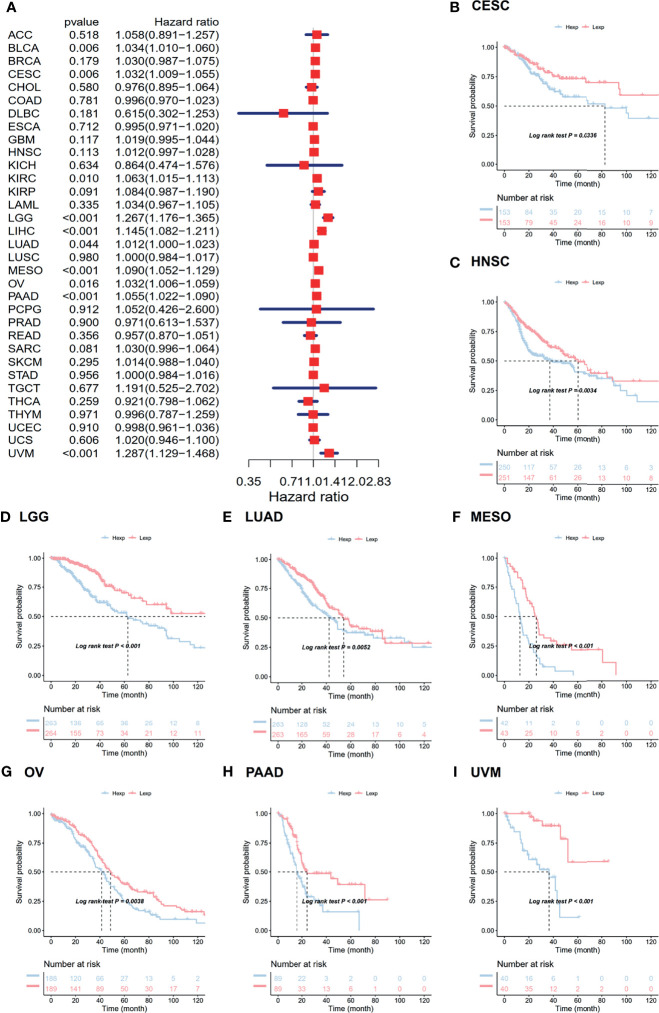
Relevance for ANXA2P2 expression levels to overall survival in months. **(A)** Forest map related to OS of pan-carcinoma. Kaplan-Meier analysis of the correlation between ANXA2P2 expression and OS in **(B)** CESC, **(C)** HNSC, **(D)** LGG, **(E)** LUAD, **(F)** MESO, **(G)** OV, **(H)** PAAD, and **(I)** UVM.

**Figure 3:**
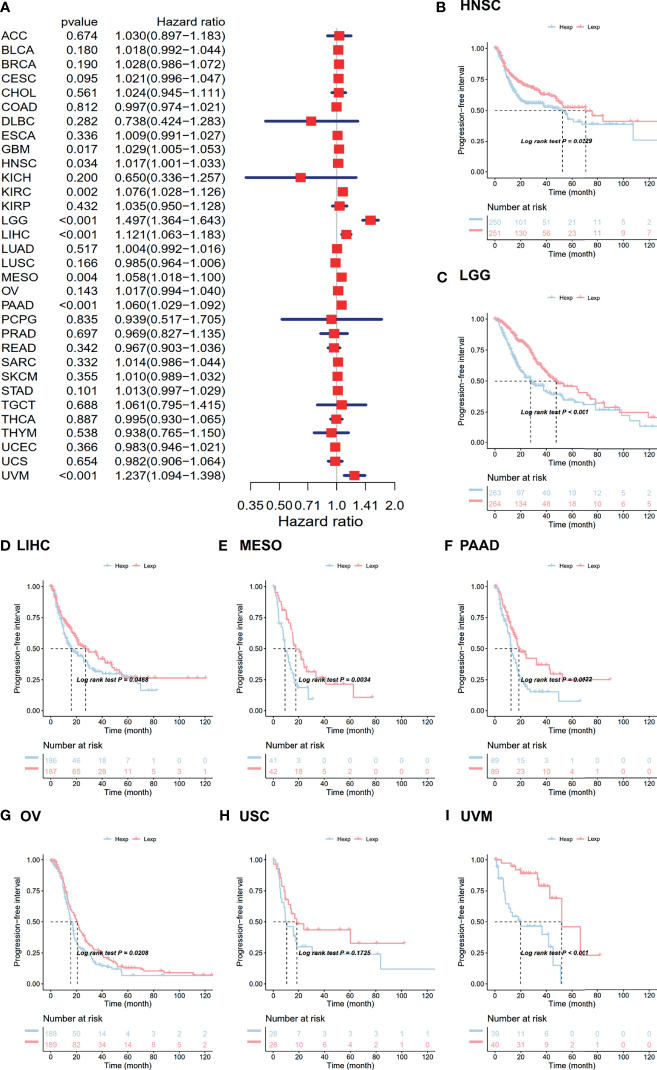
Relevance for ANXA2P2 expression levels to progression-free interval in months. **(A)** Forest map related to PFI of pan-carcinoma. Kaplan-Meier analysis of the correlation between ANXA2P2 expression and PFI in **(B)** HNSC, **(C)** LGG, **(D)** LIHC, **(E)** MESO, **(F)** PAAD, **(G)** OV, **(H)** USC, and **(I)** UVM.

The associations between ANXA2P2 expression and prognosis in multicancer patients were estimated, with survival indicators consisting of OS (**Figure 2**) and PFI (**Figure 3**). Univariate Cox regression analysis for OS indicated that ANXA2P2 expression is closely associated with OS in 10 types of cancer, including BLCA, CESC, KIRC, LGG, LIHC, LUAD, MESO, OV, PAAD, and UVM (**Figure 2A**). Additionally, KM-plot survival analysis indicated that high ANXA2P2 expression was associated with adverse OS in CESC (**Figure 2B**), HNSC (**Figure 2C**), LGG (**Figure 2D**), LUAD (**Figure 2E**), MESO (**Figure 2F**), OV (**Figure 2G**), PAAD (**Figure 2H**), and UVM (**Figure 2I**). Meanwhile, univariate Cox regression analysis for PFI suggested that ANXA2P2 transcription level was strongly related to PFI in GBM, HNSC, KIRC, LGG, LIHC, MESO, PAAD, and UVM (**Figure 3A**). KM-plot analysis manifested that high ANXA2P2 expression was relevant to adverse PFI in seven cancers, containing HNSC (**Figure 3B**), LGG (**Figure 3C**), LIHC (**Figure 3D**), MESO (**Figure 3E**), PAAD (**Figure 3F**), OV (**Figure 3G**), USC (**Figure 3H**), and UVM (**Figure 3I**). In addition, the correlation between pseudogene ANXA2P2 and its parental gene ANXA2 expression has been analyzed in **Figure S2A**, suggesting that the expression levels of pseudogene ANXA2P2 in pan-carcinoma have a strong positive correlation with its parental gene ANXA2 expression. Meanwhile, univariate and multivariate Cox regression analyses were performed on ANXA2P2 for ovarian cancer (**Figures S2B, C**), manifesting that the pseudogene ANXA2P2 can be used as an independent prognostic factor without relying on ANXA2.

### Evaluation of Tumor Immune Microenvironment

**Figure 4:**
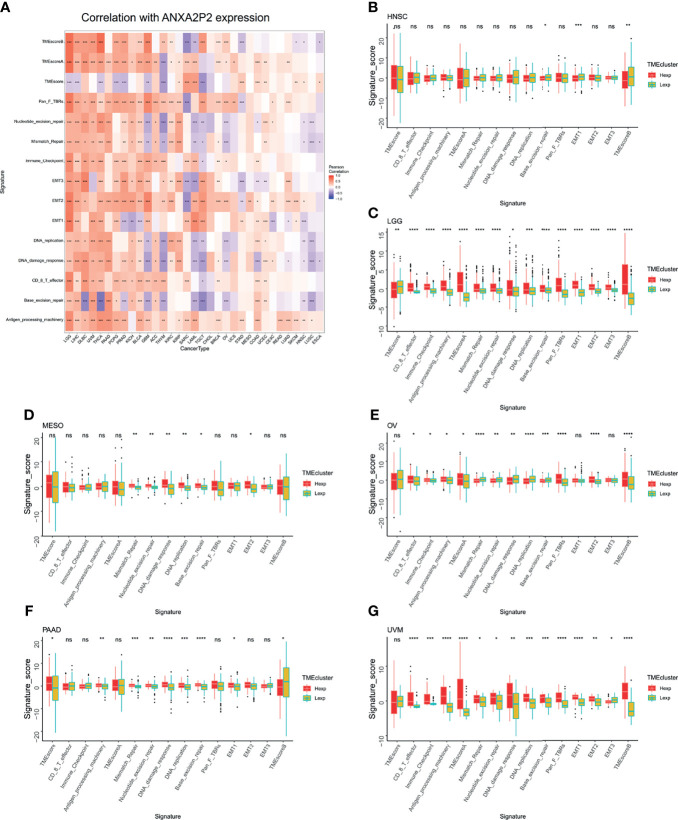
Analysis of tumor microenvironment associated with ANXA2P2. **(A)** Associations between tumor microenvironment and ANXA2P2 in pan-carcinoma. The relationships of tumor microenvironment and different ANXA2P2 expression subtypes in **(B)** HNSC, **(C)** LGG, **(D)** MESO, **(E)** OV, **(F)** PAAD, and **(G)** UVM; the red means the high-expression subtype, and the yellow-green indicates the low-expression subtype. *P < 0.05, **P < 0,01, ***P < 0.001, ****P < 0.0001 and ns indicated no significant.

Tumor microenvironment was mainly composed of tumor-related fibroblasts, immune cells, extracellular matrix, a variety of growth factors, inflammatory factors, special physical and chemical characteristics, and cancer cells themselves, which might significantly affect tumor diagnosis, survival outcome, and clinical treatment sensitivity. Therefore, *via* pan-carcinoma analysis of tumor immune microenvironment, it was discovered that ANXA2P2 expression characteristics were significantly correlated with immune microenvironment scores, nucleotide excision repair, mismatch repair, immune checkpoint, EMT, DNA replication, DNA damage response, CD8 T effector, base excision repair, and antigen processing machinery (**Figure 4A**). Next, we further explored the related scores in tumor immune microenvironment of different ANXA2P2 expression subtypes for HNSC (**Figure 4B**), LGG (**Figure 4C**), MESO (**Figure 4D**), OV (**Figure 4E**), PAAD (**Figure 4F**), and UVM (**Figure 4G**), demonstrating that mispatch repair, nucleotide excision repair, and DNA damage response scores were significantly correlated with various cancers.

### Correlation Between ANXA2P2 Expression and Immune Cell Infiltration Level in Pan-Carcinoma

**Figure 5:**
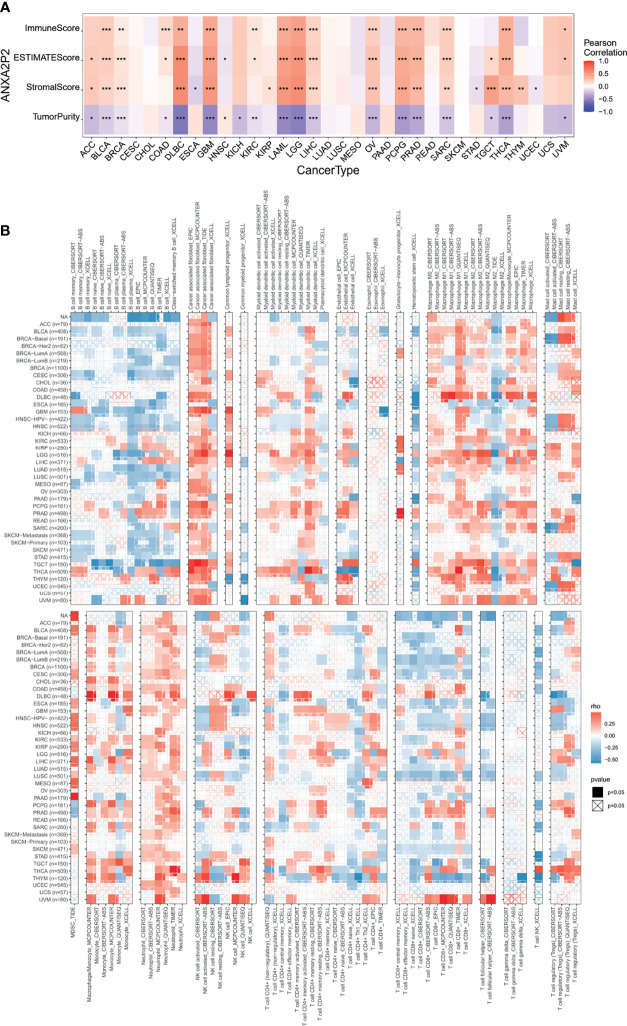
Correlation analysis of ANXA2P2 to ESTIMATE-related score and to immunocyte on the TIMER database. **(A)** The heatmap of correlation of ANXA2P2 to immune score, estimate score, stromal score, and tumor purity. **(B)** Relevance of ANXA2P2 expression to immune infiltration in pan-carcinoma. *P < 0.05, **P < 0,01 and ***P < 0.001.

To explore whether ANXA2P2 was involved in the process of immune infiltration in pan-carcinoma, the association for ANXA2P2 expression to 22 immune cell types was first evaluated based on the CIBERSORT tool. In the pan-carcinoma analysis, the transcription characteristics of ANXA2P2 were closely associated with different immune cell infiltration (**Figure S3A**), revealing that 20 cancers were significantly associated with neutrophil cells, 11 cancers were significantly associated with dendritic cells activated cells, and 9 cancers were significantly correlated with macrophage M0 cells (**Figure S3A**). Furthermore, the infiltration level of different immune cell types in HNSC (**Figure S3B**), LGG (**Figure S3C**), OV (**Figure S3D**), PAAD (**Figure S3E**), and UVM (**Figure S3F**) was also analyzed between ANXA2P2 high- and low-expression groups. Next, the relationship for ANXA2P2 expression to tumor purity, stromal score, and immune score (**Figure 5A**) was also investigated, indicating that ANXA2P2 was most significantly associated with both immune score and stromal score in DLBC, GBM, LAML, LGG, OV, PCPG, PRAD, and THCA. Also, TIMER2.0 also displayed the landscape of ANXA2P2 correlating with various immune infiltrates in pan-carcinoma *via* different algorithms (**Figure 5B**) in spite of little inconsistency among various algorithms but without too much discrepancy.

### Association of ANXA2P2 Expression Levels With TMB and MSI

**Figure 6:**
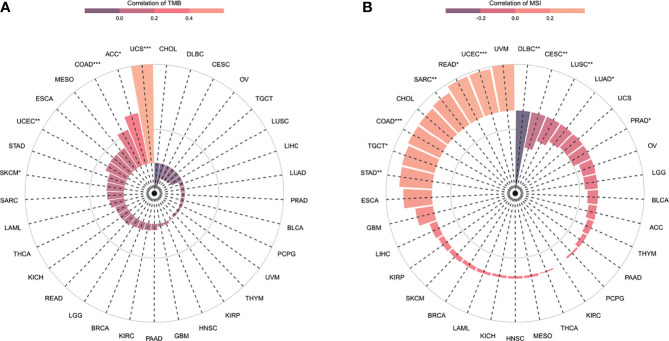
The correlation between ANXA2P2 expression and immunotherapeutic markers. **(A)** TMB and **(B)** MSI in pan-carcinoma. *P < 0.05, **P < 0,01 and ***P < 0.001.

TMB and MSI were emerging biomarkers related to immunotherapy response. So, the relationship of ANXA2P2 expression to TMB or MSI was investigated, manifesting that ANXA2P2 expression levels were significantly correlated with TMB in each tumor, including UCEC, SKCM, COAD, UCS, and ACC (**Figure 6A**) and also remarkably related to MSI in UCEC, STAD, SARC, READ, PRAD, LUSC, LUAD, COAD, CESC, TGCT, and DLBC (**Figure 6B**).

### Drug Sensitivity Analysis in Pan-Carcinoma

**Figure 7:**
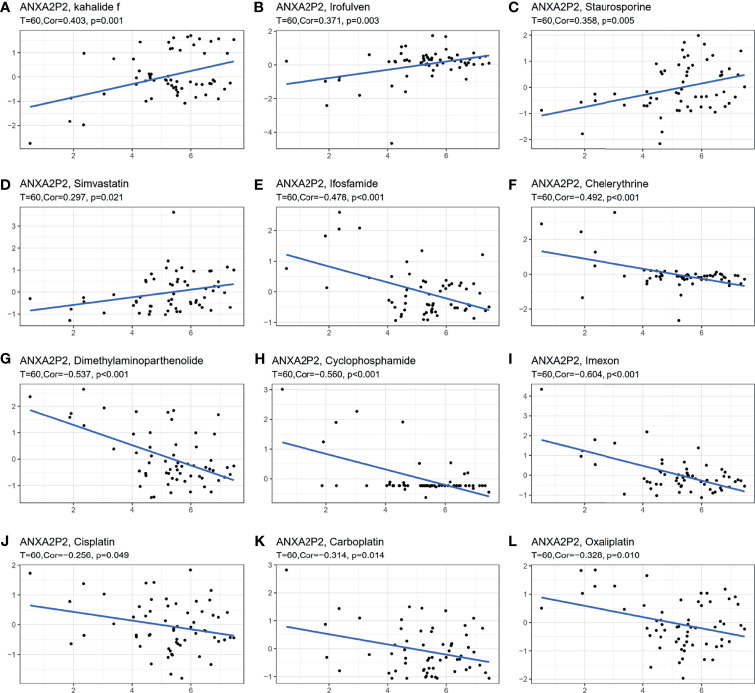
Correlation between ANXA2P2 and IC50 of drugs. **(A)** Kahalide f; **(B)** irofulven; **(C)** staurosporine; **(D)** simvastatin; **(E)** ifosfamide; **(F)** chelerythrine; **(G)** dimethylaminoparthenolide; **(H)** cyclophosphamide; **(I)** imexon; **(J)** cisplatin; **(K)** carboplatin; and **(L)** oxaliplatin.

The effect of early tumor treatment by surgery combined with chemotherapy is clear. Firstly, the sensitivity between ANXA2P2 and common antitumor drugs was explored through the CellMiner database (**Supplementary Table S1**). Secondly, the correlation between ANXA2P2 expression and drug IC50 was further calculated. Finally, we found that the prediction of high expression of ANXA2P2 was related to the tolerance of a variety of antitumor drugs (**Supplementary Table S1** and **Figure 7**). These results revealed that ANXA2P2 was notably positively correlated with kahalide F (**Figure 7A**), irofulven (**Figure 7B**), staurosporine (**Figure 7C**), and simvastatin (**Figure 7D**) and remarkably negatively related to ifosfamide (**Figure 7E**), chelerythrine (**Figure 7F**), dimethylaminoparthenolide (**Figure 7G**), cyclophosphamide (**Figure 7H**), imexon (**Figure 7I**), cisplatin (**Figure 7J**), carboplatin (**Figure 7K**), and oxaliplatin (**Figure 7L**).

### Relevance With the Key Regulatory Genes in Pan-Carcinoma

**Figure 8:**
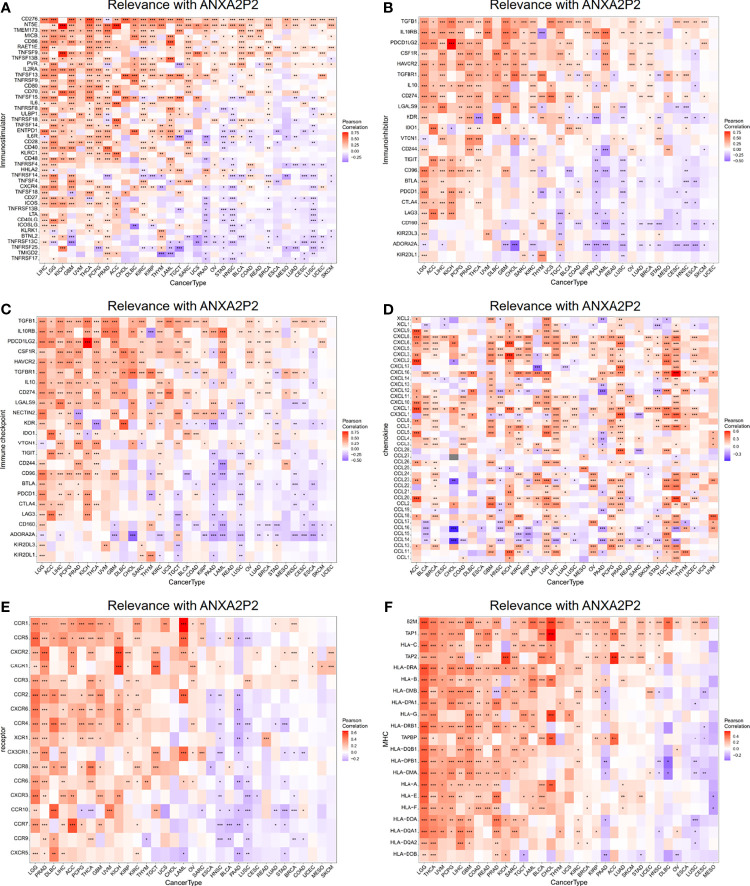
Relevance with ANXA2P2 and immune-related genes. **(A)** Immunostimulatory; **(B)** immunoinhibitory; **(C)** immune checkpoint; **(D)** chemokine; **(E)** chemokine receptor; and **(F)** MHC molecule. *P < 0.05, **P < 0,01 and ***P < 0.001.

The relationship for ANXA2P2 expression to key regulatory genes was explored using coexpression analysis in pan-carcinoma. The relevance with immune-related genes consisted of immunostimulator (**Figure 8A**), immunoinhibitor (**Figure 8B**), immune checkpoint (**Figure 8C**), chemokine (**Figure 8D**), chemokine receptor (**Figure 8E**), and MHC molecule (**Figure 8F**) indicated that almost all immune-related genes were significantly associated with ANXA2P2. In addition, ANXA2P2 had significant association with common tumor-related regulatory genes such as TNF-α signaling *via* NF-κB (**Figure S4A**), TGF-β signaling (**Figure S4B**), DNA repair (**Figure S4C**), hypoxia (**Figure S4D**), autophagy (**Figure S4E**), pyroptosis (**Figure S4F**), and ferroptosis (**Figure S4G**).

### Functional Enrichment Analysis *via* GSVA and GSEA in OV

In order to deeply investigate the molecular mechanism of ANXA2P2, we scored tumors with “gsva” and divided the samples into high- and low-expression groups by using the median of ANXA2P2 expression in OV. As shown in **Figure S5A**, the high expression of ANXA2P2 was mainly enriched in TNF-α signaling *via* NF-κB, epithelial mesenchymal transition, apical junction, IL6-JAK STAT3 signaling, cholesterol homeostasis, and inflammatory response in OV. Meanwhile, GSEA was performed to explore ANXA2P2-associated signaling pathways that were differentially activated in OV. GSEA results analyzed by KEGG indicated that ANXA2P2 is involved in base excision repair, calcium signaling, chemokine signaling, circadian rhythm-mammal, etc. (**Figure S5B**).

### Weighted Correlation Network Analysis in OV

**Figure 9:**
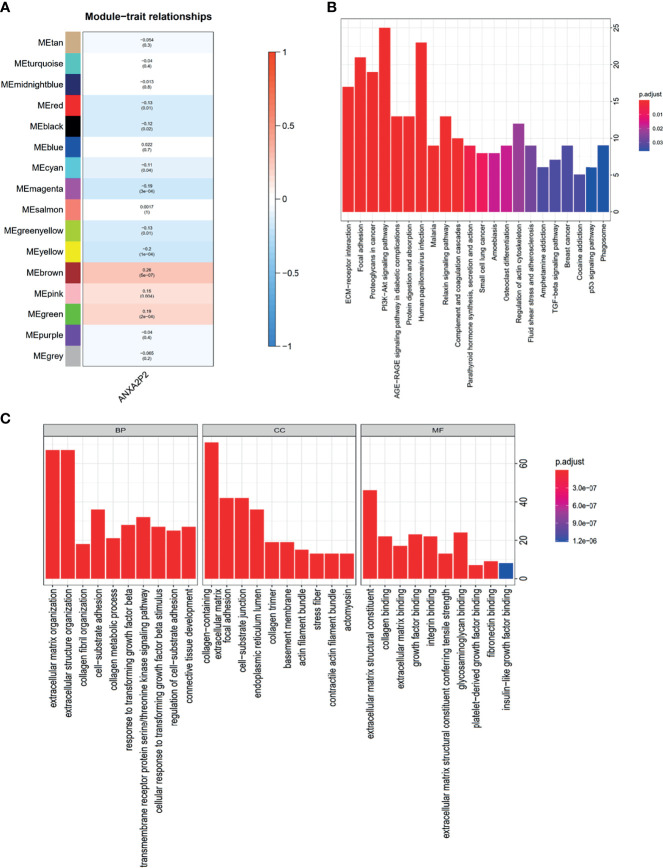
WGCNA regulatory network analysis for ANXA2P2 in OV. **(A)** Correlation between various modules and ANXA2P2 expression. The top number means the coefficient among them, and the bottom number indicates the *p*-value. **(B)** KEGG enrichment pathway analysis was applied for genes with brown module meaning highest correlation. **(C)** GO term analysis for genes with brown module meaning highest correlation.

To explore the coexpression network related to ANXA2P2, the WGCNA network was further constructed based on the transcriptional expression profile of OV. The soft threshold β was determined by the function “sft$powerEstimate” and was set to 4. Gene modules were detected according to Tom matrix and 16 gene modules were detected in this research. These gene modules were respectively black (168), blue (1,221), brown (345), cyan (77), green (310), green yellow (104), grey (142), magenta (156), midnight blue (71), pink (160), purple (115), red (171), salmon (78), tan (82), turquoise (1,485), and yellow (315) (**Figure 9A**). Further analysis between modules and traits demonstrated that brown module had the highest correlation (Cor = 0.26, *p* = 5e−07) (**Figure 9A**). Simultaneously, function enrichment analysis was performed using the brown module genes as well (**Supplementary Table S2**). The KEGG analysis indicated that genes were significantly involved in ECM-receptor interaction, focal adhesion, and proteoglycans in cancer (**Figure 9B** and **Supplementary Table S2**). GO enrichment analysis consists of biological process (BP), molecular function (MF), and cellular component (CC) analyses (**Figure 9C** and **Supplementary Table S2**). The BP analysis was mainly focused on extracellular matrix organization, extracellular structure organization, collagen fibril organization, etc. (**Figure 9C** and **Supplementary Table S2**). About CC analysis, they were notably enriched in collagen containing, extracellular matrix, focal adhesion, etc. (**Figure 9C** and **Supplementary Table S2**). Concerning the MF analysis, they were mostly involved in extracellular matrix structural constituent, collagen binding, extracellular matrix binding, etc. (**Figure 9C** and **Supplementary Table S2**).

### Association Analysis of ANXA2P2 With Core Genes and Gene Mutation Analysis in OV

To further explore the core impact of ANXA2P2 on OV, the positive (**Figure S6A**) and negative genes (**Figure S6B**) related to ANXA2P2 expression were respectively obtained, detecting that S100A10 was extremely positively related with overexpressed genes (**Figure S6A**), and HNRNPA3 and SFPQ were highly negatively relevant with overexpressed genes (**Figure S6B**). Additionally, the distribution of gene mutations was also investigated in the ANXA2P2 high/low-expression subtypes for OV. The comprehensive landscape of somatic variants visualized the mutation patterns of the top 30 driver genes with the most frequent alteration (**Figures S6C**). The significantly mutated gene mutational landscapes presented TP53 (94%), TTN (24%), and CSMD3 (9%) in OV (**Figures S6C**). These findings might provide insights into the underlying association between ANXA2P2 expression and somatic variation, thereby obtaining potential immunological and prognostic signatures in OV.

### Risk and Independent Prognostic Analysis of ANXA2P2 in OV

**Figure 10:**
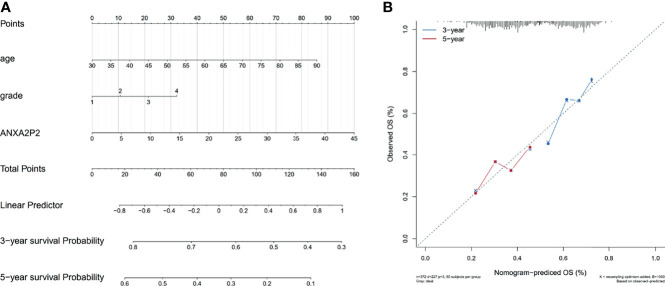
Nomogram prediction model for ANXA2P2 in OV. **(A)** The model of relevant nomogram chart, with different lines meaning different clinical features of the sample. **(B)** The corrective curve of the correlative nomogram chart, with blue for 3 years and red for 5 years.

According to the expression level of ANXA2P2 and clinical symptoms, the nomogram prediction model has been constructed and displayed in the form of nomogram for OV (**Figure 10A**). Furthermore, logistic regression analysis has indicated that ANXA2P2 expression in OV contributes greatly to the efficiency of model prediction. Simultaneously, the calibration curves for estimating survival probability at 3 and 5 years have well-matched consistency between the nomogram-predicted and observed values (**Figure 10B**), further manifesting that the nomogram prediction model is credible in predicting the prognosis of OV patients.

## Discussion

For recent years, with increasing morbidity and mortality, cancer has become the leading killer to human health (35). It is worth noting that most cancer patients in our country were already in advanced stages when they were diagnosed (36). The high incidence of cancer could not only claim great pain and death to patients but also bring heavy financial burden to their families and society (37). However, currently available therapies, including surgery, radiotherapy, chemotherapy and immunotherapy, still have limitations and cannot completely solve the dilemma. Therefore, there is an urgent need to find early diagnostic markers and effective therapeutic targets for addressing this problem.

So far, pan-carcinoma analysis has been comprehensively used to investigate the similarities and differences among various cancers, providing new perspectives for cancer prevention, early diagnosis, and treatment strategies (38–41). Simultaneously, recent studies have discovered that pseudogene ANXA2P2 has been highly expressed in a variety of tumors and played a pivotal regulatory role in the occurrence and development of tumors (42, 43). In addition, pseudogenes have been proven to have a wide range of biological functions, which could not only participate in important physiological processes such as cell differentiation, inflammation and apoptosis *in vivo*, but also affect the occurrence, development, invasion and metastasis of tumors by regulating related genes (23, 44–46).

In our study, we systematically analyzed the expression level of ANXA2P2 in pan-carcinoma and relevant normal samples, indicating that ANXA2P2 was dramatically overexpressed in a variety of cancers compared with corresponding normal tissues, including ACC, BLCA, BRCA, CESC, CHOL, COAD, ESCA, GBM, KICH, KIRC, KIRP, LGG, LIHC, LUSC, OV, PAAD, SKCM, STAD, TGCT, THCA, UCEC, and UCS on the integrated correction data of TCGA and GTEx datasets. Next, we explored the correlation of ANXA2P2 expression pattern to prognosis in a variety of tumors *via* two prognostic indicators, OS and PFI, manifesting that high ANXA2P2 expression was associated with adverse OS and PFI in HNSC, LGG, MESO, OV, PAAD, and UVM. Meanwhile, relevance between tumor immune microenvironment and ANXA2P2 was obtained, suggesting that the scores of base excision repair, DNA damage response, DNA replication, EMT, immune checkpoint, mismatch repair, and nucleotide excision repair were observably correlated with multicancer progression. The immune cell infiltration levels, including neutrophils, dendritic cells activated, macrophage M0, etc. were also remarkably relevant with ANXA2P2 in multicancer development. These results suggested that ANXA2P2 might play a pivotal part in regulating the relevant immune cells to influence tumor progression. Previous studies have shown that both TMB and MSI could be used as biomarkers to predict prognosis after immunotherapy in a variety of tumors (47–50). As an emerging and promising biomarker for tumor prediction and an important potential biomarker for immune checkpoint inhibitors, TMB and MSI may synergistically open up a new perspective for precision immunotherapy (33, 51–54). This study further revealed that the expression level of ANXA2P2 has relevance with TMB and MSI in various tumors, indicating that the expression level of ANXA2P2 would impact the TMB and MSI in many tumors, thus affecting the patient’s response to immune checkpoint inhibition therapy. Relevant results would provide a new strategy in precise immunization for multitumors. Meanwhile, drug sensitivity analysis, relevance immune regulator analysis, and pathway function enrichment also suggested that ANXA2P2 expression patterns might play an important role in the pathogenesis, development, and prognosis of various tumors.

Previously, it has been reported that high expression of pseudogene ANXA2P2 in hepatocellular carcinoma could inhibit its invasion and metastasis (43), yet high ANXA2P2 expression may promote invasive growth and metastasis of glioma cells to surrounding tissues (55, 56). Additionally, knockdown of pseudogene ANXA2P2 could significantly inhibit the progressive invasion and metastasis of glioblastoma cells *via* the PI3K/PKB pathway (42). However, relevant molecular regulatory mechanism and pathway of ANXA2P2 involved in OV development have not been elucidated so far. Therefore, we employed GSVA, GSEA, WGCNA, gene mutation, and independent prognostic analysis to explore the impact of ANXA2P2 on the tumor-related pathway, mutation site, and prognosis prediction according to its different expression patterns in OV. Subsequent results indicated that the high expression of ANXA2P2 in OV were mostly enriched in those pathways covering TNF-α signaling *via* NF-κB, epithelial mesenchymal transition, apical junction, IL6-JAK STAT3 signaling, cholesterol homeostasis, inflammatory response, etc. These pathways have been found to deal crucial acts on tumorigenesis, development, invasion, and metastasis of OV (57–62). The significantly mutated gene landscape in the high/low-expression subtypes presented TP53 (94%), TTN (24%), and CSMD3 (9%) in OV. The mutation of these genes in the biological process of tumor development has been confirmed by previous studies (63–65). The independent prognostic analysis indicated that ANXA2P2 expression level in OV contributes greatly to reliable survival prediction of 3 and 5 years. These findings might provide valuable insights into the underlying association with ANXA2P2 expression to prognosis prediction in OV.

To our knowledge, this is the first study to investigate the impact of ANXA2P2 on the pathogenesis of OV by focusing on the value of pseudogene ANXA2P2 in pan-carcinoma, and meanwhile to find new potential targets for early diagnosis and prognostic prediction of OV. Simultaneously, our study may lay the foundation for prospective functional researches and may provide new strategies for the clinical treatment of OV, thus with subsequent new hope for OV patients.

## Data Availability Statement

The original contributions presented in the study are included in the article/**Supplementary Material**. Further inquiries can be directed to the corresponding author.

## Author Contributions

YZ and XC conceived the study. XC supervised the whole project. YZ and TD performed the data curation and analysis. YZ wrote the manuscript. XC participated in the manuscript editing and discussion. All authors listed have made a substantial, direct, and intellectual contribution to the work and approved it for publication.

## Funding

This investigation was supported by the Chinese Postdoctoral Science Foundation (2020M673232) and Post-Doctor Research Project, West China Hospital, Sichuan University (2020HXBH115).

## Conflict of Interest

The authors declare that the research was conducted in the absence of any commercial or financial relationships that could be construed as a potential conflict of interest.

## Publisher’s Note

All claims expressed in this article are solely those of the authors and do not necessarily represent those of their affiliated organizations, or those of the publisher, the editors and the reviewers. Any product that may be evaluated in this article, or claim that may be made by its manufacturer, is not guaranteed or endorsed by the publisher.
